# Dr. Ludwig Schroeter

**Published:** 1912-04

**Authors:** 


Dr. Ludwig Schroeter of Buffalo died last month in Capri, Italy, having left home a few weeks previously in the hope of regaining his health. He was born in Poland in 1860, graduated in medicine at Berne, Switzerland in 1889 and shortly after came to Buffalo. He was especially well known as an obstetrician, being consultant at St. Mary's Maternity Hospital, the Buffalo General and the German Hospital. He was a member of various medical societies. It is perhaps significant of Dr. Schroeter's attachment for the land of his adoption, that his remains will be brought to Buffalo. It is no betrayal of professional confidence to speak of the quiet heroism displayed by Dr. Schroeter during the insidious progress of his last illness, especially in the face of circumstances particularly trying to a patient educated in medical matters,—the negative results of investigation along lines at first suggesting themselves to the patient: the gradual development of other symptoms, the ordeal of an operation at a distance from home, the search for health in the face of grave doubts as to its possibility.

